# Whole-Genome sequencing in routine Mycobacterium bovis epidemiology – scoping the potential

**DOI:** 10.1099/mgen.0.001185

**Published:** 2024-02-14

**Authors:** Adrian Allen, Ryan Magee, Ryan Devaney, Tara Ardis, Caitlín McNally, Carl McCormick, Eleanor Presho, Michael Doyle, Purnika Ranasinghe, Philip Johnston, Raymond Kirke, Roland Harwood, Damien Farrell, Kevin Kenny, Jordy Smith, Stephen Gordon, Tom Ford, Suzan Thompson, Lorraine Wright, Kerri Jones, Paulo Prodohl, Robin Skuce

**Affiliations:** 1 Agrifood and Biosciences Institute, Veterinary Sciences Division, Belfast, UK; 2 Queen’s University Belfast, school of Biological Sciences, UK; 3 Department of Agriculture, Environment and Rural Affairs for Northern Ireland, Belfast, UK; 4 Central Veterinary Research Laboratory, Kildare, Ireland; 5 University College Dublin, Dublin, Ireland

**Keywords:** Bovine tuberculosis, genome epidemiology

## Abstract

*Mycobacterium bovis* the main agent of bovine tuberculosis (bTB), presents as a series of spatially-localised micro-epidemics across landscapes. Classical molecular typing methods applied to these micro-epidemics, based on genotyping a few variable loci, have significantly improved our understanding of potential epidemiological links between outbreaks. However, they have limited utility owing to low resolution. Conversely, whole-genome sequencing (WGS) provides the highest resolution data available for molecular epidemiology, producing richer outbreak tracing, insights into phylogeography and epidemic evolutionary history. We illustrate these advantages by focusing on a common single lineage of *M. bovis* (1.140) from Northern Ireland. Specifically, we investigate the spatial sub-structure of 20 years of herd-level multi locus VNTR analysis (MLVA) surveillance data and WGS data from a down sampled subset of isolates of this MLVA type over the same time frame. We mapped 2108 isolate locations of MLVA type 1.140 over the years 2000–2022. We also mapped the locations of 148 contemporary WGS isolates from this lineage, over a similar geographic range, stratifying by single nucleotide polymorphism (SNP) relatedness cut-offs of 15 SNPs. We determined a putative core range for the 1.140 MLVA type and SNP-defined sequence clusters using a 50 % kernel density estimate, using cattle movement data to inform on likely sources of WGS isolates found outside of core ranges. Finally, we applied Bayesian phylogenetic methods to investigate past population history and reproductive number of the 1.140 *M*. *bovis* lineage. We demonstrate that WGS SNP-defined clusters exhibit smaller core ranges than the established MLVA type - facilitating superior disease tracing. We also demonstrate the superior functionality of WGS data in determining how this lineage was disseminated across the landscape, likely via cattle movement and to infer how its effective population size and reproductive number has been in flux since its emergence. These initial findings highlight the potential of WGS data for routine monitoring of bTB outbreaks.

**Impact Statement**

The use of classical molecular epidemiology has enhanced our understanding of bovine tuberculosis (bTB) in Britain and Ireland, showing that the wider epidemic is typically constituted as a series of spatially clustered micro-epidemics dominated by common genotypes. This has bolstered the hypothesis that ‘local’ effects play an important role in disease epidemiology and enabled identification of potentially linked outbreak cases. Based as they are on typing of only a few genetic loci however, these classical methods lack the resolution to facilitate fine scale disease tracing. Whole genome sequencing (WGS) provides the highest resolution possible for outbreak tracing and brings with it the ability to perform robust, time stamped evolutionary analyses that can reveal more about how disease has spread historically and pathogen population dynamics through time. In this manuscript, we demonstrate these advantages by comparing classical genotyping to WGS for a common *Mycobacterium bovis* lineage in Northern Ireland. Recently, much work has focused on using WGS to elucidate transmission dynamics in complex, multi-host bTB outbreaks. There are perhaps more quotidian uses that have been overlooked. We demonstrate to policy makers and field veterinarians that WGS can aid with routine monitoring of outbreaks and tracking impacts of disease eradication measures.

## Data Statement

All sequence data have been deposited in the European Nucleotide Archive - specifically BioProjects PRJEB65421, PRJNA925930, PRJEB9025.

Code and BEAST XMLs used in the production of this manuscript can be found at the link below:

https://github.com/AdrianAllen1977/Whole_Genome_Sequencing_in_Routine_Mycobacterium_bovis_Epidemiology_Scoping_the_potential.

Locations of cattle herds have been removed from Supplementary Data (available in the online version of this article) to protect personal data.

## Introduction

The use of molecular markers to discriminate genetically-related pathogen types (variants) and to inform on population structure, phylogeography and epidemiology of *Mycobacterium bovis* has a long pedigree [1]. Spoligotyping [2, 3] and Multi-Locus Variable number of tandem repeat Analysis (MLVA) have, until recently, been the molecular tools of choice for these applications [1, 4–7] and have revealed the striking clustering of molecular types that has revealed the wider bTB epidemic is a series of spatially constrained micro-epidemics. The latter feature has enabled analyses of population-level epidemiological questions of direct policy relevance, as well as providing a valuable track-and-trace functionality to outbreak investigation.

The undoubted usefulness of these methods for inferring potential epidemiological links between cases has, however, been historically compromised by their comparatively low resolution [8] and susceptibility to homoplasy [9]. By comparison, whole-genome sequencing (WGS) of pathogens provides the highest resolution available for pathogen surveillance [10]. By analogy, if spoligotyping and MLVA are like comparing different editions of the same book by reading every hundredth word, WGS is like a thorough editor reading every letter. Such increased resolution is already helping to improve epidemiological inferences for a variety of human and animal pathogens, not least, *M. bovis* [11].

Recently, much effort has been dedicated to the use of WGS to inform on transmission dynamics within the *M. bovis* epi-system in a variety of international locations [12–20]. The specific goal in these studies has been to try to unpick the Gordian knot of the relative importance of intra- and inter-species transmission in complex, sympatric, multi-host livestock and wildlife populations. The latter has obvious utility for policy makers seeking to make informed decisions for the management and monitoring of disease eradication schemes and indeed, much has been learnt, not least that there may be no single paradigm for *M. bovis* transmission dynamics, which appears to vary depending on spatial and temporal scales.

*M. bovis*, like other members of the *Mycobacterium tuberculosis* complex (MTBC) is a slowly-evolving pathogen (0.15–0.5 substitutions per genome per year in studies above). WGS of outbreaks on short temporal scales therefore, tend to exhibit considerable genetic homogeneity [20]. This is a general feature of bacterial pathogens when compared to faster evolving viruses, which can leave a more substantial trail of mutations to facilitate finer scale disease tracing [21]. The polytomies that result from such genetically homogeneous, short temporal window outbreak data can affect the robustness of the Bayesian phylogenetic trees used to infer transmission dynamics [20, 22] and it is therefore accepted that in such settings genomic data from MTBC organisms can have relatively limited utility [23, 24]. However, it is evident that when phylodynamic methods are successfully applied to *M. bovis*, this is best enabled by using multiple samples from the same locale, collected from multiple hosts over a wide temporal window of 10–30 years [12–20]. This facilitates detection of robust molecular clock behaviour, which is essential for inferring transmission dynamics [25, 26]. This is, of course, predicated on having access to systematically-sampled and highly structured archives of bacterial culture or sequence data from multiple host species over a sufficiently wide temporal window at various spatial scales – something which may not always be feasible, at least in the short term.

There is, however, a wider translational use for WGS data beyond attempts to infer local transmission dynamics. Tracing sources of infection with improved precision is of obvious benefit for veterinarians ‘on the ground’ and can be facilitated using less structured datasets. Similarly, such datasets, albeit still with some temporal range, can facilitate investigations into historical changes in population size, which can aid understanding of epidemic history, potentially informing and monitoring future disease control interventions.

In this preliminary study using a relatively small number of *M. bovis* genomes, we report on the application of WGS to a small number of widely dispersed Northern Irish isolates from a common lineage of *M. bovis,* defined by a single spoligotype and MLVA genotype/spoligotype combination. We have previously observed that, owing to the clonality of *M. bovis,* these common MLVA/spoligotype variants (genotypes) represent ancestral nodes deep in the pathogen’s phylogeny [20, 27], defining monophyletic lineages that can be further characterised by WGS. Here, we contrast the utility of MLVA/spoligotyping methods and WGS for disease tracing within one such common lineage. For outbreak investigation, we exploit the observation of geographical localisation (core range) by pathogen type and the WGS-based relatedness of isolates (phylogeny).

Specifically, for one *M*. bovis lineage, we seek to assess the difference in resolution of phylogeographic core ranges built using classical MLVA/spoligotpying versus WGS data, and to demonstrate the usefulness of the latter for outside and within core range disease source tracing. Furthermore, we seek to demonstrate how WGS data of this continuously evolving lineage, unlike discrete MLVA / spoligotype data, facilitates time stamped Bayesian phylogenetic analyses. We seek to demonstrate how the latter can enhance epidemiological investigations by informing on historic disease spread across landscapes and also how pathogen effective population size and epidemic reproductive number change over time.

## Methods

### Sampling

From an archive of MLVA genotyped and spoligotyped bovine tuberculosis (bTB) herd breakdown data [7], we selected 2108 records of one of the most commonly-observed and widely-dispersed molecular types (MLVA type 1, spoligotype SB0140; 1.140) found in Northern Ireland (NI) over the period 2000–2022. Specifically, we selected bovine home-bred animals with the single disclosing isolate (the first isolate from the herd) representing the whole breakdown (*n*=1,981) and road traffic accident (RTA) badgers (*N*=127) [28]. Both homebred cattle and philopatric wildlife like badgers produce bacterial isolates which come from animals more likely to be embedded in locales that are dominated by their locally endemic lineage of *M. bovis*, which permits more robust definition of home and core ranges.

We then created a subset of all the available genome sequenced isolates of the 1.140 genotype, with wide geographic distribution across NI over that period. We used two data sources. From previous *M. bovis* WGS studies (EBI bioproject PRJEB9025 and NCBI bioproject PRJNA925930), we selected 29 whole-genome sequences of isolates of the 1.140 molecular type; in addition, we selected 119 isolates from our archive which are publicly available as EBI bioproject PRJEB65421.

The 148 WGS isolates came from a variety of hosts – Badger *N*=27; Bovine *N*=115; Deer *N*=1; Otter *N*=1; Ovine *N*=4. Sample meta-data for both data sets are presented in Supplementary Data 1.

**Fig. 1.**
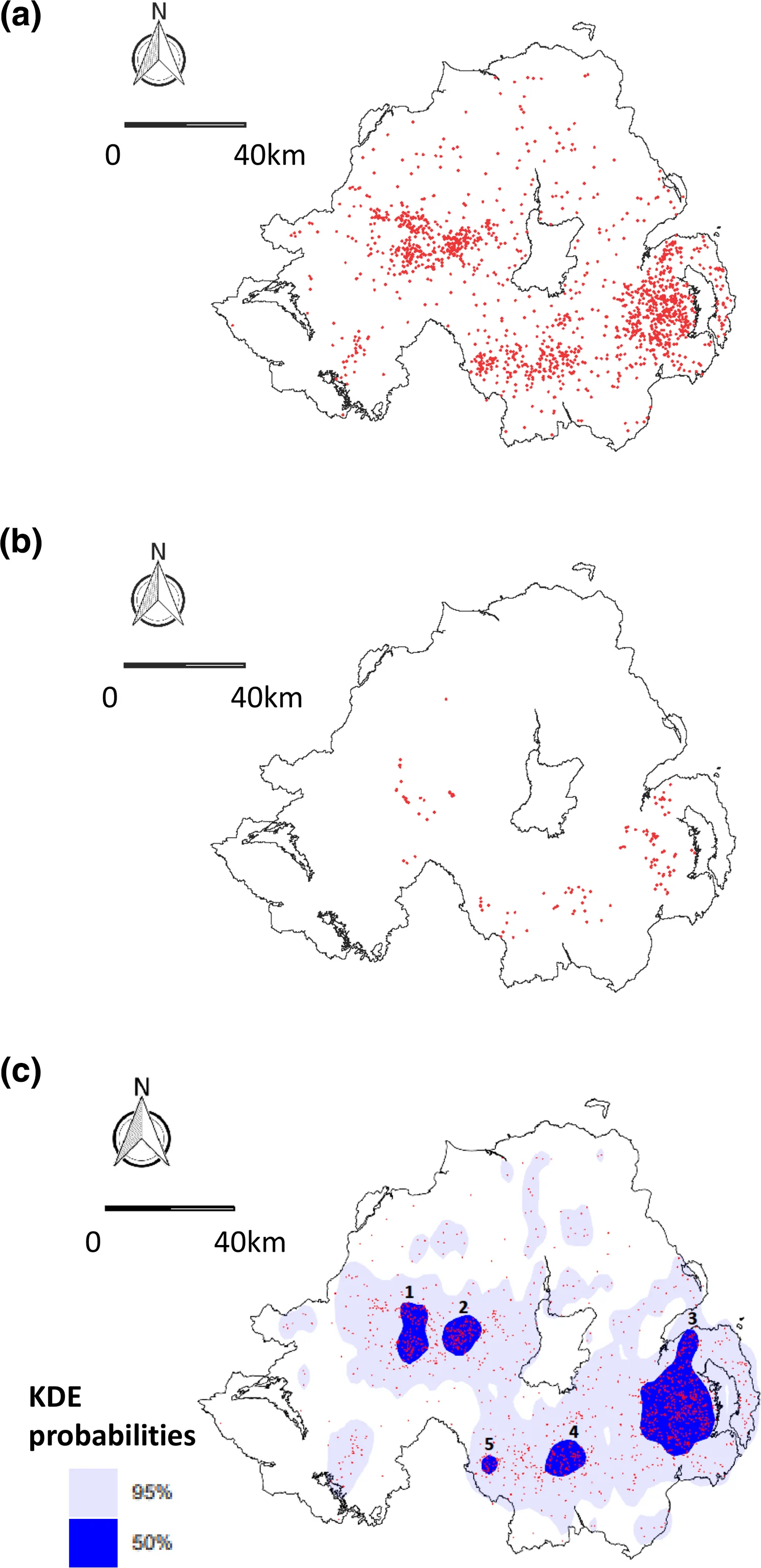
a: Geolocations of herd-level, homebred cases of bTB breakdowns (*n*=2,108) caused by the *M. bovis* MLVA-spoligotype 1.140 molecular type collated over the period 2000–2022. **b**: Geolocations of the down-sampled subset of 1.140 isolates subjected to WGS (*n*=148), collated over the period 2000–2022. **c**: As per map A, geolocations of 1.140 MLVA-spoligptype isolates and 95 and 50% kernel density estimates of ‘home’ and ‘core’ ranges.

Locations of the 2108 home-bred, spoligotype and MLVA-typed isolates are shown in Fig. 1a. Locations of the 148 WGS-sample subset are shown in Fig. 1b.

### *M. bovis* culture and DNA extraction

*M. bovis* isolates were initially cultured in the liquid BD BACTEC MGIT 960 system before sub-culture to solid Stonebrinks and Löwenstein-Jensen media, where single colonies were selected for sub-culture [4]. Isolates were heat-killed in a water bath at 80 °C for 30 min before extracting DNA using standard high salt/cationic detergent cetyl hexadeycl trimethyl ammonium bromide (CTAB) and solvent extraction protocols [29, 30].

### Spoligotyping and MLVA analysis

All *M. bovis* isolates were genotyped by spoligotyping [2] and 8-locus MLVA using previously described methods [6]. >Authoritative names for spoligotype patterns were obtained from https://www.mbovis.org/ [3]. MLVA profiles were named using an in-house laboratory nomenclature, based originally on the frequency of surveyed isolation [4].

### WGS and bioinformatics

The selected 119 isolates were whole-genome sequenced as follows. Sequencing libraries were prepared using the Illumina DNA Prep method to produce inserts of approximately 500–600 bp and sequenced at AFBI using the Illumina MiSeq platform with Illumina V2 chemistry, producing paired-end reads of 250 bp.

Reads for all samples were mapped to the annotated reference genome for *M. bovis* isolate AF2122/97 (GenBank accession LT708304.1) [31] using the mapping-based phylogenomics pipeline, RedDog V1beta.10.3 [32]. Alignment and mapping were carried out by Bowtie2 v2.2.9 [33] using the ‘sensitive local’ mapping setting. SNP calling was undertaken using SAMtools and BCFtools [34] using the consensus caller setting. A minimum depth of 10 x was set for SNP calling. The average coverage failure filter, average depth filter and average mapping failure filters were set at 98 %, 10 x, and 80 %, respectively. Transposable elements, repeat regions and the PE/PPE regions, as defined in the Genbank annotation, were excluded from SNP calling using the parseSNP tool in the RedDog pipeline [32]. We also re-sequenced a locally-held culture of the *M. bovis* reference genome, AF2122/97, as a positive quality assurance control.

#### Phylogenetics and clade definition

We assessed the most appropriate nucleotide substitution model for our phylogenetic analysis using the ‘modelTest’ function of the package ‘Phangorn’ [35] in R [36]. Specifically, we assessed the fit of the General Time Reversible (GTR), Jukes Cantor (JC), and Hasegawa Kishino Yano (HKY) models to our SNP alignments. The nucleotide substitution model with the lowest Akaike Information Criterion (AIC) (GTR – AIC:5902.02; HKY – AIC:5913.50; JC – AIC:6146.57) was used to build a maximum likelihood phylogeny using RAxML-ng with the autoMRE rapid bootstrapping search method selected and stopped after 3000 replicates [37] and the phylogeny was visualized in ggtree in R [38]. We accounted for ascertainment bias by including a correction for the number of constant sites observed using the method recommended in the RAxML manual [37].

We defined clades/clusters of closely related isolates in the resulting tree using a 15 SNP cut-off (SNP15 hereafter). A variety of SNP cut-offs have been used to identify clusters of potentially linked TB cases. In the United Kingdom (UK), SNP5 and SNP12 clusters have been proposed for *M. tuberculosis* [39] while in other studies SNP10 thresholds have been proposed for *M. tuberculosis* and *M. bovis* [15, 40–43]. Meehan *et al*. [8] suggest that SNP distances of between 1 and 5 in *M. tuberculosis* can be consistent with transmission occurring up to ten years in the past. Choosing an optimal cut-off can therefore be somewhat subjective. We used SNP15, as per the example of van Tonder *et al*. [17] as it allows for incorporation of older bTB transmission events and also accounts for any variance in the rates of mutation amongst the sampled isolates. To apply the SNP15 cutoff to our RAXML tree, we used the Python tool ‘TreeCluster’ v 1.0.3 [44]. Specifically, we used the Max cluster function to define clades for which all members exhibited a maximum distance between all isolates of, at most, 15 SNPs. To investigate within-clade potential epidemiological links, we also assessed a 5 SNP cut-off in the same function (see below).

### Mapping and home and core range estimation

We chose to define home ranges and core ranges using the 95 and 50 % kernel density estimate (KDE) that ecologists have used for a variety of animal species [45, 46]. We mapped the MLVA/spoligotype and WGS datasets (15 SNP cut-off) in R using ‘ggplot2’ [47] and used the function ‘geom_hdr’ from the package ‘ggdensity’ [48] to calculate the 50 and 95% KDE for mapped locations. For the spoligotype and MLVA dataset, we used the method of Sheather and Jones [49] to determine the best value for bandwidth/smoothing parameter (h) in the production of kernels – the Sheather and Jones approach is reported to work well for large numbers of samples which exhibit a multimodal distribution [50]. For the SNP15 WGS data sets, we only mapped clades defined by TreeCluster that had at least eight member isolates, with only one isolate per farm location. We calculated the areas of the 50 % KDE polygons for all mapped clusters using the packages ‘sf’ [51] and ‘*sp*’ [52]. We used the default Silverman’s rule of thumb to calculate the bandwidth as it is reported to outperform the Sheather and Jones method for small numbers of samples (<100) [50].

### Disease tracing - outside core range cases

For WGS isolates, which mapped outside of the 50 % core range KDE of their SNP15 defined clade, we accessed the NI Animal and Public Health Information System (APHIS) [53] to investigate whether the disclosing animal, or conspecifics at the outlying location, had a registered movement, which could be traced back/close to the ‘core range.’

### Outbreak investigation within core range

Interestingly, one of the SNP15-defined clades, with a tightly clustered core 50 % KDE (see below in results section ‘Disease tracing – ‘outside core range’ cases’**,** clade four results), comprised isolates from multiple host species – badger *N*=4; bovine *N*=11; ovine *N*=4; otter *N*=1. We applied a 5 SNP cut-off to this clade using ‘TreeCluster v 1.0.3’ [44] to investigate potential epidemiological links between isolates.

### Bayesian phylogenetics – molecular clock testing

For all Bayesian phylogenetic analyses, we used ascertainment bias correction by accounting for the number of invariant / constant sites across the *M. bovis* genome.

Before undertaking any Bayesian phylogenetic analyses, we assessed whether the 1.140 WGS dataset exhibited a temporal signal, conservatively, using the programme TempEst v1.5.1 [54]. After finding the the best-fitting outgroup root (Reference genome AF2122/97), the root-to-tip divergence model was fitted using the residual mean squared method.

To test the significance of the temporal signal more robustly in our dataset, we randomised the tip dates for the 148 selected isolates, in ten replicates, using the method of Firth *et al*. [25] for a constant population, strict clock model and a skyline, strict clock model. We randomised tip dates using the ‘Tipdatingbeast’ package [55] in R. Replicates for both models were run in BEAST 2 (Bayesian Evolutionary Analysis by Sampling Trees) [56] using the GTR nucleotide substitution model and a chain length of 200 000 000 MCMC steps. We discarded 10 % of these steps as burn-in. We checked convergence in Tracer 1.7.1 [57] (ESS>200 for all parameters). Following the removal of a 10 % burn-in, chains were combined using LogCombiner v2.6 [56].

### Bayesian phylogenetics – inferring evolutionary rates, effective population size dynamics and reproductive number of the 1.140 lineage

We opted to use several Bayesian phylogenetic models to inform us on the epidemiology of the 1.140 lineage – specifically we used a standard constant population size, coalescent based approach alongside the Bayesian Skyline [58] and Birth Death Skyline (BDSky) [59] approaches. All models can infer the timing of diversification of clades, which when combined with geographic location data can tell us something about how the disease has spread across the landscape. In addition to this, the Skyline and BDSky models can tell us something about the change in bacterial population size through time, epidemic reproduction number through time and the mean time of being infectious – crucial information for field veterinarians to be able to access when running eradication schemes. For all three types of model we opted to use both strict and relaxed lognormal clocks. A relaxed clock has previously been suggested to be the optimal approach for slowly evolving bacterial pathogens [21], however our experience in the past has been that on rare occasions outputs from analyses can vary if a strict clock is used. So, in this case we used both to assess if any of the inferred clade timings and epidemiological parameters were affected by clock model choice.

Specifically, we performed equivalent analyses to those described above for non-randomised tip dates: six models were run in Beast2 [56]: using (1) constant population size assumptions with strict and relaxed log normal clocks; (2) skyline [58] variability in population size with strict clock and relaxed lognormal clocks; (3) birth death skyline (BDSky) [59] variability in epidemic growth and contraction with strict clock and lognormal clocks. Analytical parameters and approaches were as discussed above for randomized tip analyses. XML files for these analyses are in the Supplementary Files.

We compared the median substitution rate for all randomised and non-randomised models to assess the temporal signal in the data.

Maximum Clade Credibility (MCC) trees were constructed for combined chains using TreeAnnotator v2.6 [56] using the median ancestor heights criterion.

## Results

### WGS

The selected 119 *M*. *bovis* isolates produced good quality sequence reads in line with the criteria on coverage, depth and mapping specified in the Methods section. Alongside the 29 previously sequenced isolates, mean coverage across the AF2122/97 reference genome was 99.01 % (s.d. 7.83), mean depth 51.55 (s.d. 37.64) and mean % of reads mapping to reference 97.98 % (s.d. 9.54). Mean base quality scores were 34.5 (s.d. 1.14); see Supplementary Data 2 for details. The RedDog pipeline identified 378 SNPs in this sample, which passed all quality filters across all samples compared to reference. Genome locations of all SNPs relative to AF2122/97 are detailed in Supplementary Data 3. The re-sequenced AF2122/97 reference strain was observed to exhibit a 3 SNP distance from the sequence archived at Genbank. We have noted this difference before [20] and assessed that it is probably due to diversification in the cultures with the accrual of new SNPs happening over the ~20 year period we have had the reference strain in our freezer, occasionally being broken out in fresh cultures to facilitate its use as a control.

### Phylogenetics and clade definition

**Fig. 2.**
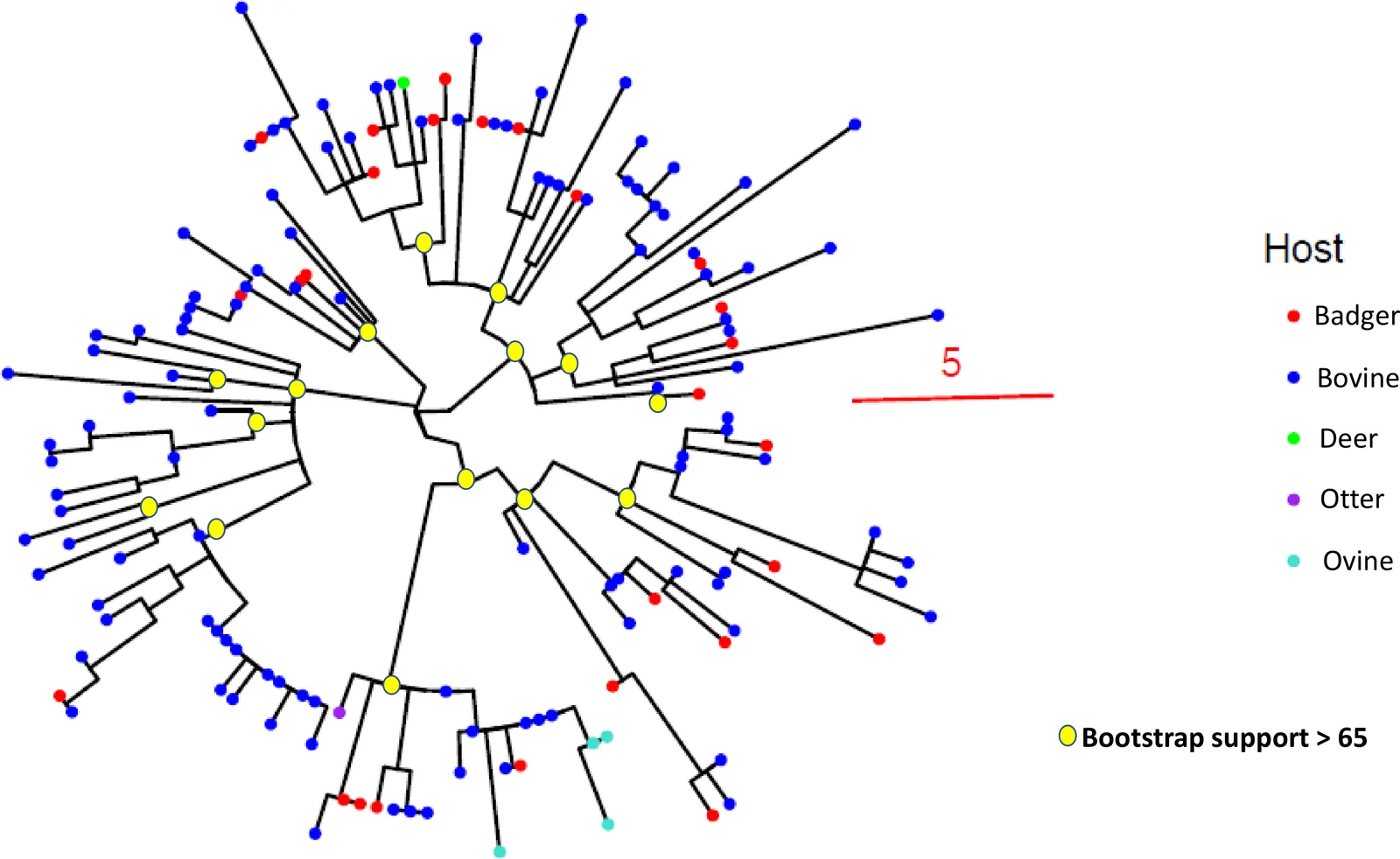
378 SNP maximum likelihood phylogeny of WGS 1.140 (n=148) isolates. Scale bar in red is no. of SNPs. Hosts that *M. bovis* was isolated from are colour coded at the tips.

The 148 isolate, 378 SNP maximum likelihood phylogeny produced by RaxML-ng is shown in Fig. 2, rooted against the reference sequence AF2122/97 (removed to aid visualisation). Major nodes exhibited bootstrap support greater than 65 % (Fig. 2) and mean, pairwise, inter-isolate SNP distance was observed to be 13.79 SNPs (s.d. 4.27).

**Fig. 3.**
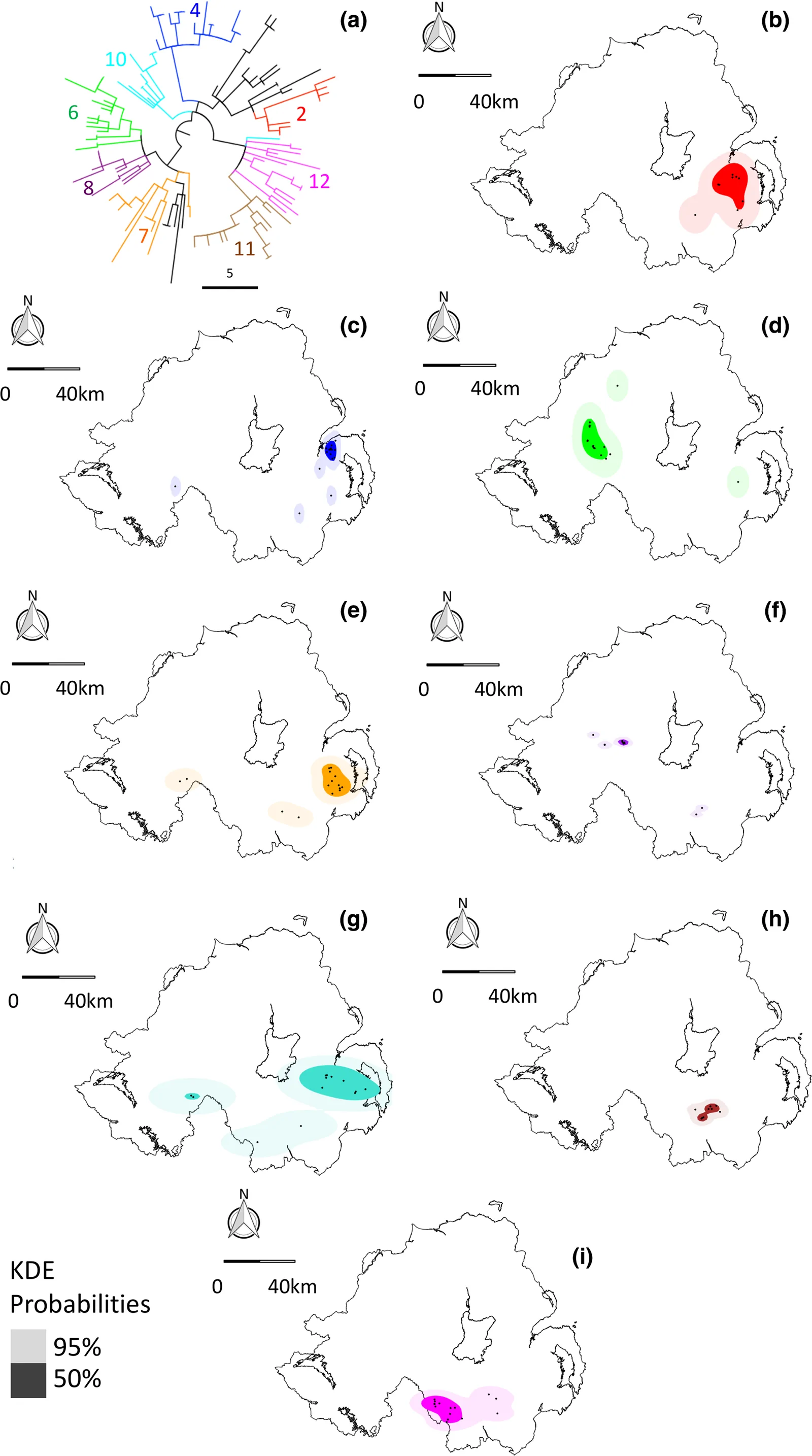
a: Maximum likelihood phylogeny with SNP15 clades numbered and colour coded to link to home range maps. Scale bar at bottom of phylogeny is no. of SNPs. **b**: Clade 2 95 and 50% KDE home and core ranges. **c**: Clade 4 95 and 50% KDE home and core ranges. **d**: Clade 6 95 and 50% KDE home and core ranges. **e**: Clade 7 95 and 50% KDE home and core ranges. **f**: Clade 8 95 and 50% KDE home and core ranges. **g**: Clade 10 95 and 50% KDE home and core ranges. **h**: Clade 11 95 and 50% KDE home and core ranges. **i**: Clade 12 95 and 50% KDE home and core ranges.

The TreeCluster SNP15 threshold identified 12 clades within this phylogeny. Eight of 12 clusters from the SNP15 cut-off contained at least eight isolates from unique farms and accounted for 124(84 %) of the 148 isolates; these are colour coded in Fig. 3a.

Conservative assessment of a temporal signal using Tempest indicated a significant, positive relationship between root-to-tip SNP distance and year of isolation (Fig. S1A – r^2^=0.37, slope=0.55 SNPs per year, *P*<0.01).

### Mapping and core range estimation

The MLVA.spoligotype 1.140 homebred, herd-level cases exhibited a wide distribution across NI (Fig. 1a). Five 50 % KDE core ranges were observed, with a combined area of 961.5 km^2^ (Fig. 1c and Table 1). A breakdown of area by each of the localised core ranges is presented in Table 1.

**Table 1.** 50 % KDE core range areas for the spoligotype and MLVA typed and the WGS SNP15 cutoff datasets. Clades are colour coded to aid reference to Fig. 3

Mlva 1.140	Area km^2^	No. samples in clade
50 % KDE total	961.5	n/a
50 % KDE area 1	149.1	n/a
50 % KDE area 2	115.5	n/a
50 % KDE area 3	561.9	n/a
50 % KDE area 4	111.3	n/a
50 % KDE area 5	23.7	n/a
**WGS 15 SNP cut off**		
Clade 2 50 % KDE	321.4	9
Clade 4 50 % KDE	43.5	16
Clade 6 50 % KDE	216.2	17
Clade 7 50 % KDE	228.8	16
Clade 8 50 % KDE	16.2	12
Clade 10 50% KDE	660.9	13
Clade 11 50 % KDE	67.9	9
Clade 12 50 % KDE	227.8	15

The clades delineated by SNP15 mapped to geographically clustered core ranges across NI (Fig. 3b–i). Areas of the 50 % KDE core range for each of these colour coded clusters from SNP15 phylogenies are presented in Table 1. These WGS SNP-defined core ranges were considerably smaller than the combined 50 % KDE core range observed for the 1.140 molecular type – ranging from 16.2 to 660.9 km^2^ (Table 1).

### Disease tracing – “outside core range” cases

Clade two exhibited two bovine isolates that mapped outside of the 50 % KDE core range (Fig. 3b). The first of these outlying isolates was found on a farm just on the southern edge of the core range (two kilometres (km)) and also originated from a farm with a natal herd close to the core range (one kilometre from its edge). The second outlier passed through a herd within the core range via trade before being detected as a reactor in its new herd within an area previously described by Akhmetova *et al*. [20] in the southeast of NI.

Clade four contained five bovine isolates which mapped outside of the 50 % KDE core range (Fig. 3c). All four bovine isolates to the south and west of the core range could be traced back to within it from trade movements. The other outlying bovine isolate found just north of the core range lacked a movement linkage but was only 1.6 km from the range edge.

Clade six exhibited three isolates which mapped outside of the 50 % KDE core range (Fig. 3d). The most northerly outlier from a bovine animal exhibited no obvious links back to the core area. The most easterly outlier came from a badger isolated in 2008, the earliest collected isolate in the clade. This isolate’s location is close to the core range of clade 7, which shares close ancestry with clade 6 (Fig. 3a). We suspect clade 6’s western core range was seeded by animal movement from an area in the east of NI and that this outlying badger isolate may come from the region from which that seeding event occurred. The remaining outlying isolate was found only 1.2 km from the core range edge.

Clade seven exhibited five bovine isolates which mapped outside of the 50 % KDE core range (Fig. 3e). Two of these were found in the south-eastern region of NI that was the subject of the recent study by Akhmetova *et al*. [20] and could be linked back to the core range by cattle movements. One isolate due east of the core range was found only 3 km from its edge on the shore of Strangford Lough. The remaining two outliers were found in the west of NI in County Tyrone. The most westerly isolate could be linked back to the core range as the affected animal had in its lifetime passed through a herd 1.5 km from its edge before disclosing infection at its new location. The remaining County Tyrone based sample had no obvious links back to the core range.

Clade eight exhibited two small 50 % KDE core ranges in close proximity – 9 km apart – in the centre of County Tyrone (Fig. 3f). Three bovine isolates mapped outside of these 50 % KDE core ranges (Fig. 3f). The two bovine outliers found in the southeast of NI were from the study area previously examined by Akhmetova *et al*. [20]. No obvious link back to the core range was found for these isolates. The remaining outlier, and most westerly of isolates from clade eight also lacked an obvious link back to the core range.

Four isolates, three bovine and one badger, from clade 10 mapped outside the main eastern 50 % KDE core range (Fig. 3g). The outlying cattle isolate immediately to the south of the core range, in the area previously studied by Akhmetova *et al*. [20], could be linked back to a natal herd close to the core range (0.4 km from its edge). The most southerly outlier, a cattle isolate could not be linked back to the core range. Two outliers were observed in the west of NI in County Tyrone, one a bovine isolate and the other a badger isolate. The bovine isolate could be traced back to a natal herd in the core range and the badger isolate was found only 1.5 km from the farm holding in question. Furthermore, both isolates were found in 2016 and exhibited an inter-isolate SNP distance of 6 which is just outside the 5 SNP cutoff that has been used to infer being part of a potential transmission cluster. While a direct epidemiological link is perhaps unlikely owing to the latter, both isolates are in relative terms still closely genetically related and therefore likely part of the same geographically localised micro-epidemic perhaps associated with import of this lineage into this novel western region through cattle movement, and subsequent spillover to the badger population.

Two isolates from clade 11 mapped outside the 50 % KDE core range (Fig. 3h). Both outliers were only a short distance from the edge of the core range (0.6 km and 3.1 km).

Four outliers from clade 12, all from bovine animals, were observed to map outside of the 50 % KDE core ranges (Fig. 3i). The three most western outliers all lacked any movement records linking them to the core range identified. The one remaining outlier mapped to 0.5 km from the edge of the core range.

In total, 28 outside core range isolates were observed for the clades investigated. Nine of these outlier isolates (32.1 %), all from bovine animals, exhibited no obvious links back to the identified core ranges. Eight isolates (28.6 %), again all from bovine animals, exhibited direct links back to herds within the core range of their associated clade. A further nine (32.1 %) exhibited a link to a herd that was on the edge (within at least 4 km) of their associated clade’s core range. And finally, two outlier badger isolates (7.1 %) from clades 6 and 10 exhibited probable links to their clade’s core range that likely involved translocation via movement of infected cattle.

### Outbreak investigation “within core range”

**Fig. 4.**
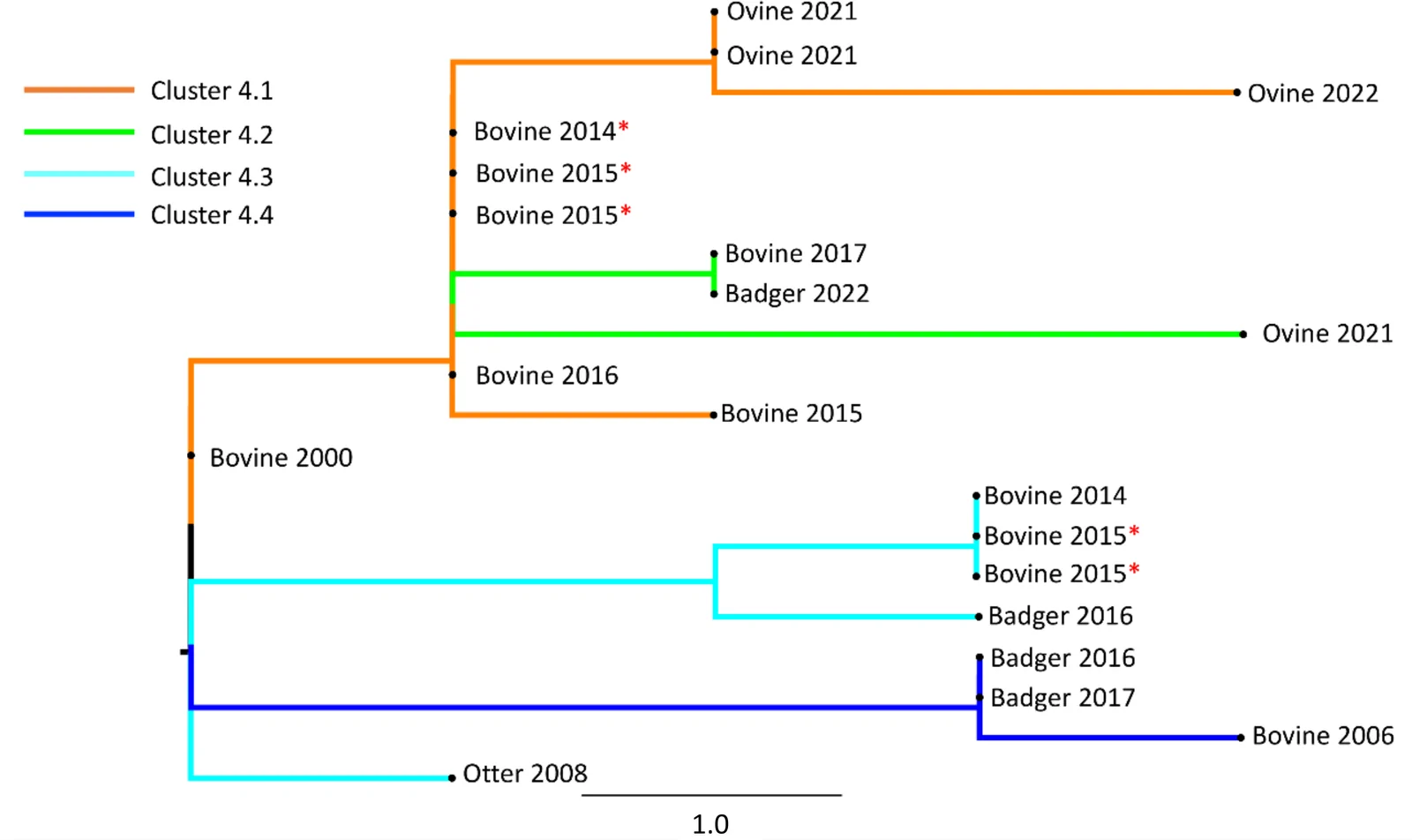
Maximum likelihood phylogeny of SNP15 defined clade 4, with SNP5 cutoff applied to identify four colour-coded, closely related, potentially epidemiologically linked clusters (4.1–4.4). Isolates marked with an asterisk were found outside the core range of clade 4. Scale bar (no of SNPS) can be found at the foot of the phylogeny.

The five SNP (SNP5 from hereon) cut-off, when applied to the 20 isolates within the SNP15-defined clade 4, identified four potentially epidemiologically-linked clusters of isolates (Fig. 4). The largest cluster (cluster 4.1) accounted for nine isolates derived from six bovines and three ovines – three of the bovine isolates were outside the 50 % KDE core range. Cluster 4.2 was closely-related to cluster 4.1 and contained three isolates from badger, bovine and ovine animals. Cluster 4.3 comprised five isolates, three from bovine animals, one from a badger and one from an otter – two of the bovines mapped outside the 50 % KDE core range. Cluster 4.4 contained three isolates, one from a badger and two from bovines.

### Bayesian phylogenetics

Tip randomisation data, for both the constant population size strict clock and skyline strict clock models, exhibited substitution rates consistently lower than those observed for non-randomised models (Fig. S1B and S1C and Table 2). The lack of overlap in 95 % highest posterior density (HPD) between randomised and non-randomised models (Fig. S1 and Table 2) is consistent with there being a significant temporal signal in the sampled 1.140 *M*. *bovis* lineage. Substitution rates (0.24–0.31 substitutions per genome per year) and ‘time to most recent common ancestor’ (tMRCA) for the four non-randomised BEAST models are presented in Table 2. The MRCA of the 1.140 clade was estimated to have been extant approximately 30–50 years before present – i.e. the early-1970s to the late 1980s.

**Table 2.** For all six BEAST non-randomised tip date models - Substitution rates (per genome per year) plus 95 % highest posterior density range; and time to most recent common ancestor plus 95 % highest posterior density

BEAST model	Substitution rate (per genome per year)	Substitution rate 95 % HPD range	tMRCA (years)	tMRCA 95 % HPD range
**Constant population strict clock**	0.26	0.20–0.32	49.3	38.3–63.4
**Constant population relaxed clock**	0.26	0.19–0.32	50.0	38.1–65.4
**Skyline strict clock**	0.24	0.17–0.32	44.3	33.4–58.9
**Skyline relaxed clock**	0.25	0.17–0.32	44.1	33.3–57.8
**BDSky strict clock**	0.31	0.25–0.37	29.7	28.2–31.2
**BDSky relaxed clock**	0.31	0.25–0.37	29.7	28.1–31.2

MCC trees for all six non-randomised tip analyses are presented in Figs S2-S7. Trees exhibited very similar topology and branching times for major clades (Table S1). The posterior supports for the nodes which are the MRCA for the eight SNP15 clades are presented in Table S2. In general, across all models, clade ancestral node support was high – typically falling between 0.63 and 1.00. An exception was clade eight whose ancestor exhibited posterior support ranging from 0.38 to 0.48. The common ancestor clade eight shared with its closest relative clade (and nearest geographic neighbour), clade 6, exhibited posterior support above 0.9 across all six models.

The southern county SNP15 clades (clusters 11 and 12 – Fig. 3h, i) in all six MCC trees diverged from a common ancestor shortly after the establishment of the whole 1.140 lineage 28–41 years before present. The eastern county SNP15 clades (clusters 2, 4, 7 and 10 – Fig. 3b, c, e, g) similarly share a common ancestor 28–42 years previous, again shortly after the emergence of the whole 1.140 lineage.

Conversely, the western county SNP15 clades (clusters 6 and 8 – Fig. 3d, f) share a later common ancestor, 20–25 years before present. In all six MCC trees, and indeed the RaxML tree in Fig. 3a, both western clades share close common ancestry with eastern clade 7, sharing a common ancestor around 23–30 years before present.

**Fig. 5.**
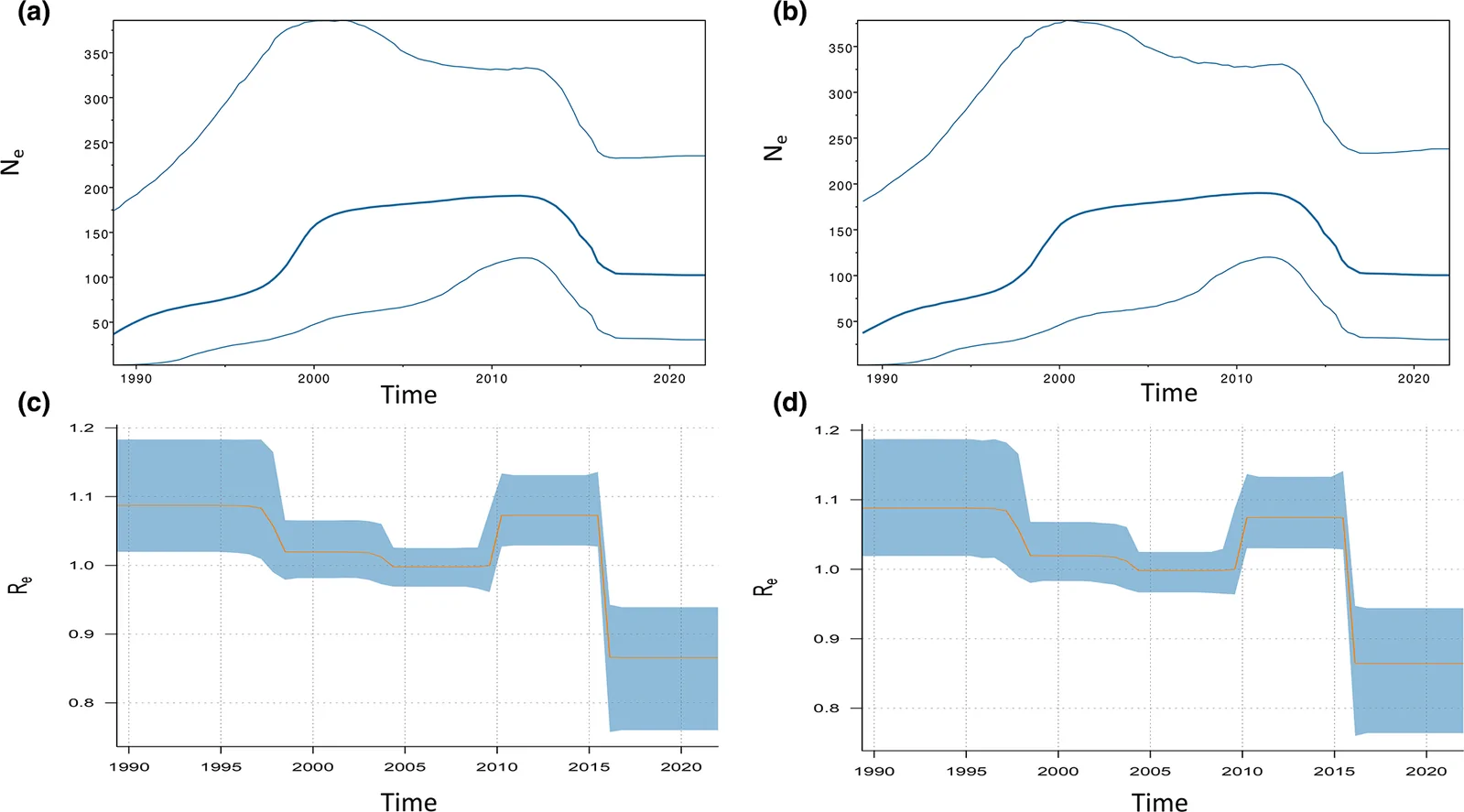
a: Strict clock Bayesian skyline plot showing how effective population size (N_e_ - dark blue line) of the *M. bovis* 1.140 lineage varies through time. Bounds of the 95% HPD of the N_e_ estimate denoted by light blue lines. b: Relaxed clock Bayesian skyline plot showing how effective population size (N_e_ – dark blue line) of the *M. bovis* 1.140 lineage varies through time. Bounds of the 95% HPD of the N_e_ estimate denoted by light blue lines. c: Strict clock Bayesian birth death skyline plot showing how reproduction number (R_e_ – dark orange line) varies through time. Light blue shaded area denotes 95% HPD of the R_e_ estimate. d**:** Relaxed clock Bayesian birth death skyline plot showing how reproduction number (R_e_ – dark orange line) varies through time. Light blue shaded area denotes 95% HPD of the R_e_ estimate.

The relaxed and strict clock skyline plotswere highly congruent, suggesting the 1.140 lineage underwent an expansion in effective population size (N_e_) throughout the 1990s, rising steeply in the latter part of that decade, peaking in the early 2010s, before undergoing a marked contraction in the period 2012–2015 [7]. Strict and relaxed clock BDSky plots of reproductive number (R_e_) through time (Fig. 5) were also very congruent and suggested that from the early 1990s to 2015 R_e_ was generally above 1, consistent with increased disease transmission in this period. From 1990–1997 median R_e_ was at is highest (1.09 95 % HPD 1.02–1.18). From 2000–2010 there was a decrease in median R_e_ (2000–2005=1.02 95 % HPD 0.98–1.06; 2005–2010=1.00 95 % HPD 0.97–1.02), but with lower 95 % HPD value observed to lie below 1. From 2010–2015 median R_e_ was observed to increase again (1.07 95 % HPD 1.03–1.13). However, after 2015, median R_e_ was observed to substantially decrease to 0.87(95 % HPD 0.76–0.94).

Both BDSky models also estimated similar median rates of becoming uninfectious (median numbers of lineages which went extinct per year) for the 1.140 lineage – strict clock 6.1(95 % HPD 3.1–10.5); relaxed clock 6.3(95 % HPD 3.2–10.8). The rate of becoming uninfectious is the reciprocal of the median time an individual is infectious for.

## Discussion

This pilot study, while based on a small number of non-randomly selected genome sequences, demonstrates the utility of WGS of *M. bovis* to aid investigation of disease outbreaks and to inform on the evolutionary history of the bovine TB epidemic in NI. Below we discuss the tangible benefits to be derived from modern genome surveillance-based approaches and outline future considerations to exploit these data more fully.

### Core ranges and epidemiological tracing outside core ranges

The wider bTB epidemic in GB and Ireland has been observed to present as a series of spatially clustered micro-epidemics, each dominated by common MLVA.spoligotypes [6, 7, 60]. This striking phylogeographic feature lends itself to epidemiological investigation – isolates retain a genetic signature which gives useful clues to their geographic origin. The exploitable ecological concept of home and core range can be applied in this context, where molecular or sequence types observed to be outside of their estimated core range(s) can often be traced back to farms and wildlife within core ranges. Consequently, we can be increasingly confident to make the distinction ‘infected when moved’ versus ‘infection acquired on arrival’. However, the home and core ranges defined by low-resolution classical MLVA/spoligotyping tend to be large and fragmented especially for the over-dispersed and more frequently isolated molecular types, often encompassing county-sized areas [6], which can limit their utility for disease tracing purposes. Here we demonstrate that higher resolution WGS data can resolve smaller core ranges to facilitate more efficient disease trace back; these smaller core ranges effectively redefine the meaning of ‘local’.

For the eight SNP15-defined core ranges we describe in this study, we find that 67.8 % of the core 50 % KDE outliers exhibited direct links to farms within, or very close to the edge of, the core range estimated for that SNP15 defined clade. There were some interesting associations which demonstrate how our current lack of data can affect inferences – in SNP15 clade 6, the major outlier from the core range was from a 2008 RTA badger (Fig. 3d), one of the earliest samples in our WGS dataset. The geographical distance of this badger from the core range is consistent with spillover following livestock movement; badgers are philopatric and do not normally tend to disperse far from natal territories [61, 62]. Two scenarios are possible – (1) it could be that a bovine moved from a western herd to a new herd in the east and brought infection that spilled over to the local badger population, or (2) it could be that the western cluster of clade six isolates was founded from an eastern location in the past where a focus of infection is found in local wildlife – see below in the Bayesian Phylogenetics section for evidence of the latter.

We acknowledge that we currently lack the number and density of randomly selected samples across the landscape to construct robust SNP-based core ranges. SNP15 clade eight is perhaps a case in point here. It exhibits a very small core range which could well expand with the addition of more samples, and incorporate previously outlying isolates. Additionally, it would be better to base home and core range construction on exclusively home-bred animals; imports, potentially reflect more distant source(s) and add noise. A complicating factor in defining home and core ranges for sequence types is the fact that the Irish agricultural landscape is considerably fragmented [63]. Farm owners can use their own land-parcels distant from home farm locations for grazing of livestock and rent distant land-parcels through the practice known as ‘conacre’ [63]. Cattle movements to distant land parcels within the same farm holding are not officially-recorded. Given that here we use the home farm location to set core ranges, we accept some degree of imprecision and systematic error has been incurred in their construction. A more appropriate geo-location may be a centroid of all land-parcels used by an affected farm [64]. As we accrue more data in future, a wide-ranging discussion on the latter, the temporal range of isolates, density of sampling and SNP cut-offs to use in defining statistically robust core and home ranges will be necessary. Setting such criteria can often appear arbitrary and vary by region/territory, but if efforts are led by the principal of maximising utility for control schemes, then there are likely substantial benefits to be realised.

Despite the shortcomings discussed, the core ranges we illustrate here were able to facilitate tracebacks of 1.140 isolates that would not have been possible previously with MLVA data. The size of the core ranges for such over-dispersed types, coupled with the density and interconnectedness of the animal trading network across Northern Ireland [65], makes finding potential epidemiologically-relevant animal movements like finding needles in county-sized ‘haystacks’. WGS data have the capacity to shrink such a ‘haystack’ down to a considerably more manageable size, thereby facilitating superior disease tracing efforts. We suggest therefore that the basic approach is fit-for-purpose and useful and could be refined by the addition of more data.

For those outside core range isolates lacking a traceback linked to recorded animal movement, it is difficult with currently available data to definitively determine their likely origin. The Northern Irish farming landscape is highly fragmented, as mentioned above [63], and there is the possibility that farmers with outlying holdings from their main farms could facilitate longer range moves of cattle without having to officially record the movement. Such behaviour could seed infection in new areas and would lack a record that facilitates definitive traceback. This said, a general rule of thumb is probably sensible here. If an outside core range occurrence is observed to be more than 10 kilometres from the edge of the core range, the likelihood badgers have been involved in the initial translocation is probably small on account of the fact badgers are largely philopatric animals [61, 62]. Therefore, it is more likely that such longer range translocations are related to cattle movement.

### Outbreak investigations within core ranges

In the SNP15 core range for clade 4 (Fig. 3a, c) the application of a SNP5 cut-off helped to identify isolates potentially linked by more recent transmission (Fig. 4). Clusters 4.1 and 4.2, while being separate after the application of the cut-off, were still closely-related; indeed the splitting of these clusters was most likely caused by the 2021 ovine sample in cluster 4.2 having a longer branch length that other isolates. These two closely-related clusters (alongside clusters 4.3 and 4.4) are consistent with sympatric cattle and badgers sharing closely-related *M. bovis* by virtue of bidirectional transmission [15, 17, 20]. The ovine isolates, which all come from the same flock, are divided across clusters 4.1 and 4.2 and are closely-related to earlier sampled bovine isolates from a neighbouring herd. The genetic distance between the ovine isolate in cluster 4.2 and those in cluster 4.1 is consistent with there being two separate introductions into the flock, possibly from sympatric infected local cattle or wildlife - ovines are generally considered to be rare, dead-end hosts for bTB [66]; this finding appears to be consistent. However, the closely-related ovine isolates in cluster 4.1 and apparent divergence in one of these isolates may be consistent with within-flock transmission. The potential for infected sheep to become a bTB source has been raised [67], but without additional WGS sampling of sympatric, infected cattle it is difficult to be more confident. The otter from cluster 4.4 is testament to the wide host range of *M. bovis* [68, 69].

The homogeneity of isolates from within core range outbreak settings admittedly makes inference of ‘who infected whom’ using genomic data alone impossible for such a slowly evolving pathogen as *M. bovis* [20]. However, the information on genetic relatedness that WGS data provides, can guide outbreak investigations to narrow the potential sources of infection as we have demonstrated here. With the addition of other meta-data relating to livestock movement and land parcel use it may be possible to refine these investigations further [18].

### Bayesian phylogenetics - Inferring past population history and epidemiological parameters of lineages

While most of the practical ‘on the ground’ utility for *M. bovis* genome epidemiology is described in the section above, there are additional, richer insights that can be gleaned from the information content of whole-sequence data that have the potential to improve, uniquely, our understanding of the history of outbreaks. We suggest that such insights may be useful for policy makers, researchers and veterinarians involved in disease outbreak investigations.

Our Bayesian phylogenetic analyses identified a robust temporal signal in our data of ~0.24–0.31 SNP substitutions per genome per year, which is consistent with the evolutionary rate described for *M. bovis* in previous studies [13, 14, 20, 70]. The presence of such a signal permits us to make robust inferences on the timing of ancestral nodes and past population demography which can help characterise the epidemic and its spread. The posterior support for the majority of SNP15 defined clade common ancestors was high which gives us confidence in the inferences we have made (Table S2). In the case of the western clade eight which had low support, we were able to use the ancestor it shares with clade 6 (also part of the western 1.140 micro-epidemic) to time emergence of the western focus of infection. The ancestor had a much higher posterior support across all six Bayesian phylogenetic models, which again gives us confidence in our findings.

All six BEAST models suggested the tMRCA for the 1.140 lineage occurred ~30–50 years before present. Skyline plots (Fig. 5a, b) for our 1.140 lineage are consistent with the general increase in prevalence seen throughout the 1990s. The decrease in effective population size (N_e_) observed in the early to mid-2010s is also consistent with systematic MLVA surveillance data, which suggested the 1.140 molecular type decreased in frequency in this period, going from the most common genotype observed in Northern Ireland, to the second most common [4, 6, 7]. To observe this frequency decrease previously required detailed surveillance over many years, with hundreds of spoligotype and MLVA typed samples – with WGS data we can infer the same trend directly from genomic diversity data from only 148 samples collected over a similar time frame.

Interestingly, the BDSky analyses of how R_e_ varied with time (Fig. 5c, d), were largely consistent with the coalescent based skyline analyses. The general observation that disease transmission of the 1.140 lineage, as measured by R_e_, was at its highest in the early to late 1990s correlates with the observed increase in N_e_ over that same period. The subsequent decrease in R_e_ to ~1 from 2000 to 2010 is also congruent with the plateauing of N_e_ observed over the same period. The sharp decrease in R_e_ from 2015 onwards also tallies with the observed decrease in N_e_ over the same timeframe. It is encouraging these tools can track the progression of disease transmission dynamics using a small number of genomes over time. The fact these data are supported by extensive animal level MLVA data over the same time period is useful validation of genomic tools. We do not have any firm leads on why the 1.140 lineage has experienced a contraction in population size. A feature of bTB incidence in many territories is an unexplained ‘saw-tooth’ style graph through time as unexplained rises and falls occur against the background of ongoing control schemes.

From the the BDSky estimates for median rate of becoming uninfectious (6.3 95 % HPD 3.2–10.8) we could estimate mean period of time of being infectious. The latter is the reciprocal of the rate and is a combined measure of infected animals being removed from the epi-system by detection and removal, natural death and recovery. The mean infectious period for the 1.140 lineage was observed to be 2 months but with a skewed 95 % HPD suggesting this could be anywhere between 1–4 months. The latter is very similar to that observed in the recent work of Akhmetova *et al*. [20] and suggests that detection of infection for the majority of animals is likely to fall within the first year of being infected, which is congruent with known diagnostic test performance characteristics [71, 72] and NI’s control scheme which tests all bovine animals at least once annually [73]. The time-stamped Bayesian MCC trees (Figs S2-S7) also help us to investigate the broader, historic dissemination of the 1.140 genotype across Northern Ireland in a way that spoligotype and MLVA data alone cannot do. From all four BEAST models, it is apparent that the extant southern and eastern clades of the 1.140 lineages diverged around the same time after the MRCA, expanding and diversifying 30–40 years before present to occupy the remarkably stable foci of infection they maintain today, despite extensive control efforts [6, 7]. Interestingly, all models suggest the western clade was founded in the last 20–23 years by *M. bovis* bacteria sharing ancestry with established eastern 1.140 clades, and in particular clade 7 (Fig. 3e). The latter is suggestive that cattle movement from the east of NI likely resulted in the establishment of this novel focus of infection. A further interesting link is that as discussed above, a 2008 badger isolate from Clade six is the only ‘outside core range’ case found in the east of NI. This badger isolate is also the earliest collected sample we have from clade 6. It could be that rather than being linked by a long range movement of cattle from the west to east and a spill-over into the badger population, the badger isolate actually represents a currently under-sampled sub-population residing in the area of eastern NI that seeded infection into the novel western focus. The close ancestry of clades 6 and 7 and the finding that the badger’s location is in the clade seven core range provide further evidence of this scenario. More broadly, the observation that longer range moves of cattle are most likely associated with the wider dissemination of bTB across landscapes is consistent with the findings of a recent phylodynamic analysis of multiple bTB epi-systems in GB, which found the latter were the primary mechanism by which infection was translocated to new regions [17].

These evolutionary approaches, designed to tell us something of the history and demography of pathogens, are not just of academic interest. They may be useful for policy makers and field veterinarians wishing to undertake trend monitoring or targeting of specific lineages of *M. bovis* within the wider epidemic. When applied to single lineages or sub-lineages, perhaps within defined geographic areas undergoing specific control interventions, these methods could retrospectively inform on long term trends related to increased transmission, expansion of core ranges and the effectiveness of control schemes. All these events will leave genetic signatures which can be detected, and which could be used to target specific outbreaks more effectively.

Currently the type of retrospective analyses of outbreaks described above are the most likely use of WGS for epidemiology. The latter is a feature forced on us by the biology of the organism. It takes 56 days to culture *M. bovis* isolates for genome sequencing, and in the interim many field veterinarians will have completed their initial disease outbreak investigations. New methods to bypass culture and produce good quality genomic data straight from clinical isolates are coming [74], and will in all likelihood make ‘real time’ genomic epidemiology possible, but until they are routinely used and quality assured to produce high quality outputs, the retrospective approach we outline is the best currently available.

### Defining nomenclature

An important consideration for modern, pathogen genome epidemiology is nomenclature. Unlike classical MLVA/spoligotyping methods with discrete classification categories, WGS pathogen data gathered over time changes our paradigm to working with dynamic, measurably evolving pathogens [21]. Naming evolving lineages, and the daughter lineages they produce, took on special significance during the SARS-CoV-2 pandemic [75, 76]. Such dynamic nomenclature uses strings of numbers to define the lineages and sub-lineages an isolate belongs to and is expandable as new lineages evolve. Similar naming conventions have been proposed for the M. tuberculosis Complex, including *M. bovis* [77]. A criticism of such schemes, however, has been that while useful for computational biologists and epidemiologists, they do not translate well to ‘on the ground’ practitioners who must undertake outbreak investigations. Having a standardised nomenclature that can facilitate data intensive epidemiological analyses and have meaning for field veterinarians will be necessary. Future efforts should be made to harmonise these outcomes.

### Conclusion

We show that WGS data have considerable functionality and practical utility for aiding our understanding of, and control of, bTB. Our data are preliminary and based on a small number of non-randomly-selected samples. Despite this, we show the disruptive potential of whole-genome surveillance for disease tracing, outbreak investigation and long-term trend monitoring of pathogen lineages. More work needs to be done to define the sampling frames, SNP thresholds, data visualisation and standardised nomenclature that can shape and facilitate ‘best practice’ in the use of genomic tools for this and other ‘wicked problem’ pathogens, but we believe herein we provide the evidence and impetus to encourage such future discussions and work.

## Supplementary material

10.1099/mgen.0.001185 Uncited Supplementary Material 1.

10.1099/mgen.0.001185 Uncited Supplementary Data Sheet 1.

## Abbreviations

AIC: akaike information criterion
APHIS: animal and public health information system
BDSky: birth death skyline
BEAST: bayesian evolutonary analyses by samping trees
bTB: bovine tuberculosis
bTB: bovine tuberculosis
GTR: general time reversible
HKY: Hasegawa Kishino Yano
HPD: highest posterior density
JC: jukes cantor
KDE: kernel density estimate
MCC: maximum clade credibility
MGIT: mycobacterial growth indicator tube
MLVA: multi locus VNTR analysis
MRCA: most recent common ancestor
MTBC: *Mycobacterium tuberculosis* complex
N_e_: effective popualtion size
NI: Northern Ireland
R_e_: reproduction number
SARS-CoV-2: severe acute respiratory syndrome coronavirus 2
SNP: single nucleotide polymorphism
tMRCA: time to most recent common ancestor
VNTR: variable number of tandem repeats
WGS: whole genome sequencing
