# Supplementary material for: Whole-Genome sequencing in routine Mycobacterium bovis epidemiology – scoping the potential
Source: Microb Genom. 2024 Feb 14;10(2):001185. doi: 10.1099/mgen.0.001185 (PMC10926703; doi:10.1099/mgen.0.001185)
Supplement: Uncited Supplementary Material 1. [file mgen-10-01185-s001.pdf]

Supplementary Figures and Tables

Whole-Genome Sequencing in routine *Mycobacterium bovis* epidemiology – scoping the potential.

Adrian Allen[1]*, Ryan Magee[2], Ryan Devaney[1], Tara Ardis[1], Caitlín McNally[1], Carl McCormick[1], Eleanor Presho[1], Michael Doyle[1], Purnika Ranasinghe[1], Philip Johnston[3], Raymond Kirke[3], Roland Harwood[3], Damien Farrell[4,5], Kevin Kenny[4], Jordy Smith[5], Stephen Gordon[5], Tom Ford[1], Suzan Thompson[1], Lorraine Wright[1], Kerri Jones[1], Paulo Prodohl[2], Robin Skuce[1].

1. Agrifood and Biosciences Institute, Veterinary Sciences Division, Belfast, UK.
2. Queen's University Belfast, school of Biological Sciences, UK.
3. Department of Agriculture, Environment and Rural Affairs for Northern Ireland, Belfast, UK.
4. Central Veterinary Research Laboratory, Kildare, Ireland.
5. University College Dublin, Dublin, Ireland.

*Corresponding author

Adrian.Allen@afbini.gov.uk

Keywords:

Bovine tuberculosis, genome epidemiology

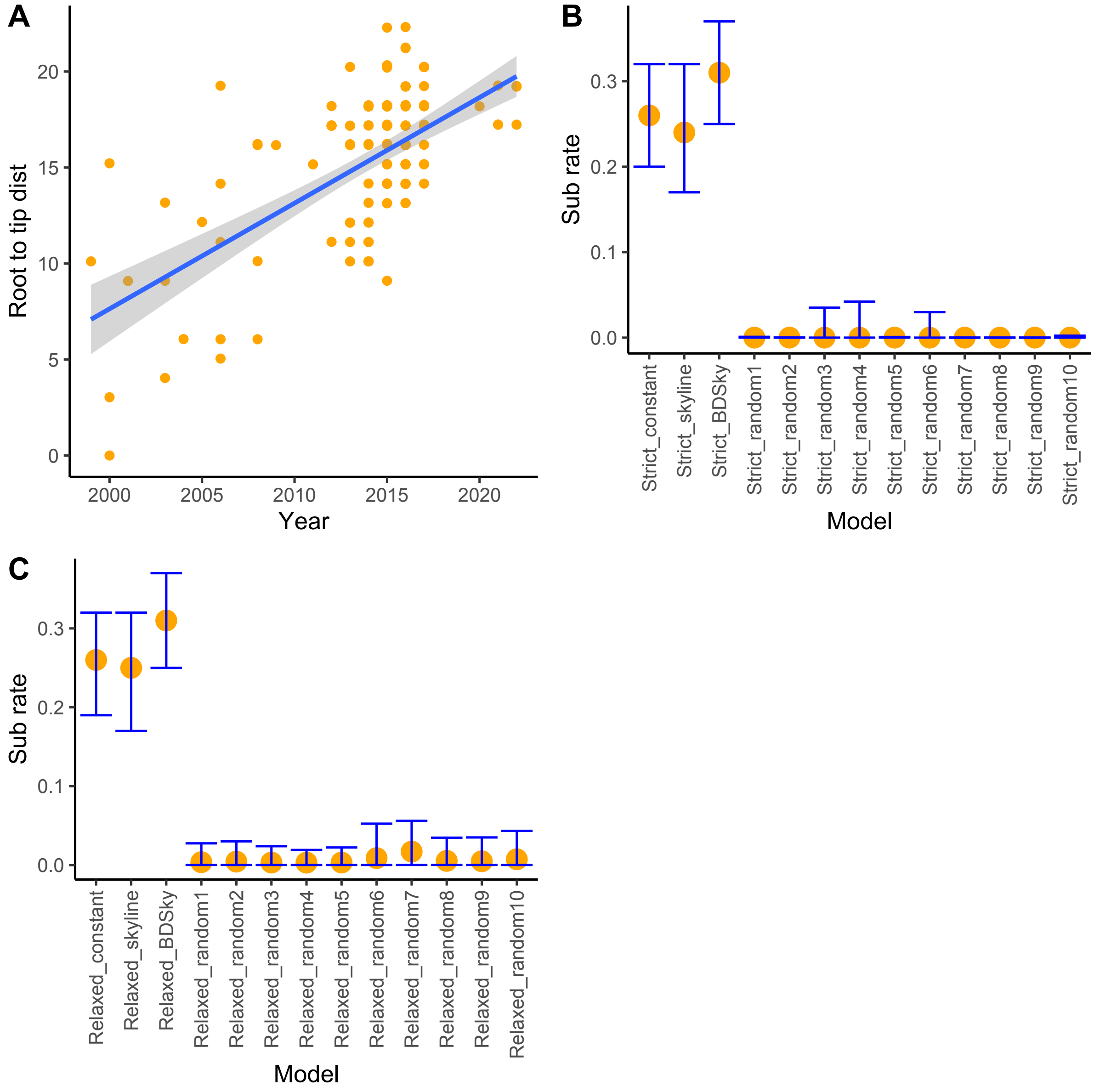

Supplementary Figure S1 – Temporal signal plots. **A**: Conservative assessment of signal in Tempest – $p<0.001$, slope 0.55 substitutions per genome per year, r^2 0.37. **B**: Non-randomised strict clock constant population and skyline model substitution rates versus substitution rates from ten tip randomised strict clock subsets. **C**: Non-randomised relaxed clock constant population and skyline model substitution rates versus substitution rates from ten tip randomised relaxed clock subsets.

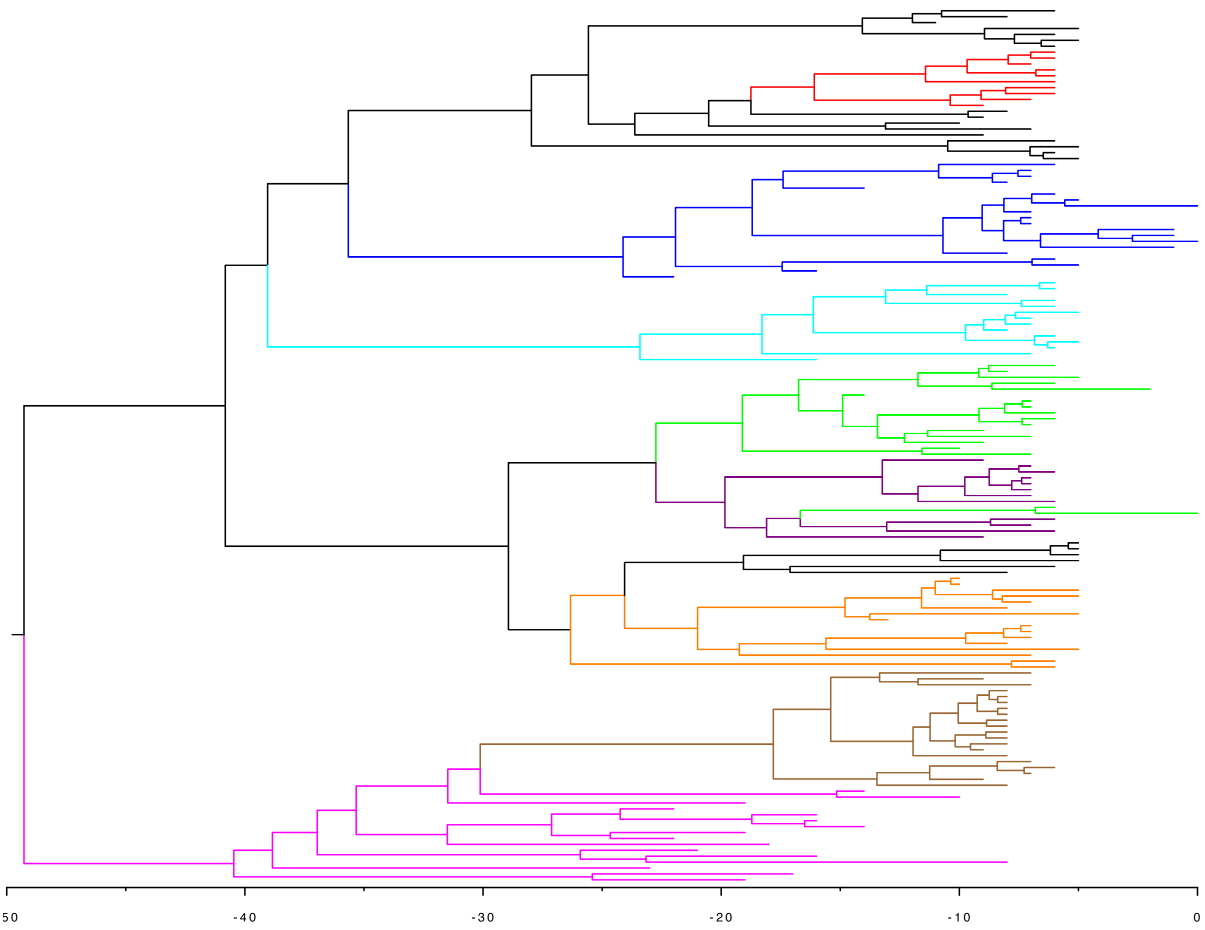

Supplementary Figure S2 – Constant population size, strict clock, MCC tree. NI 1.140 lineage 15SNP clusters 2 (red), 4 (blue), 6 (green), 7 (orange), 8 (purple), 10 (turquoise), 11 (brown) and 12 (pink) - colour coded as per figure 3A.

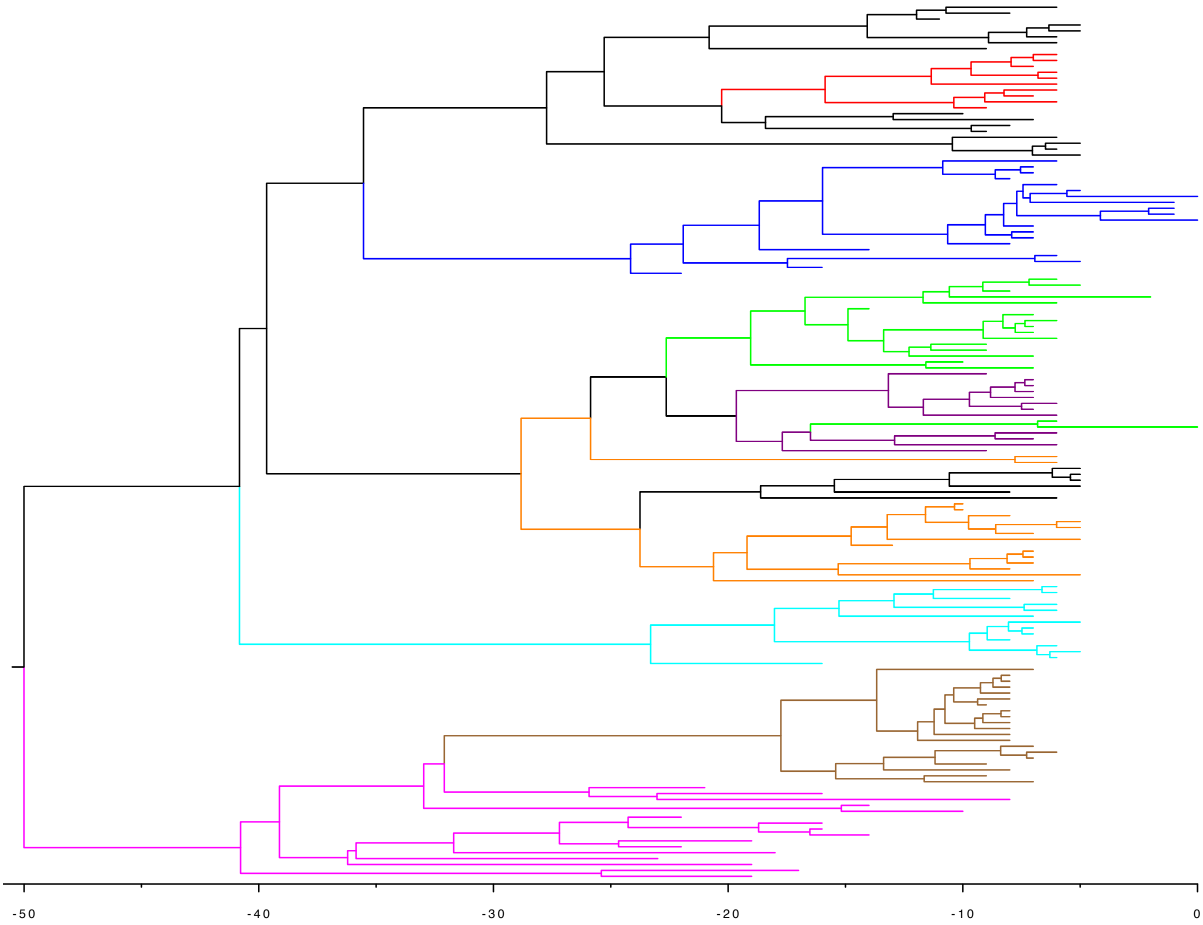

Supplementary Figure S3 – Constant population size, relaxed clock, MCC tree. NI 1.140 lineage 15SNP clusters 2 (red), 4 (blue), 6 (green), 7 (orange), 8 (purple), 10 (turquoise), 11 (brown) and 12 (pink) - colour coded as per figure 3A.

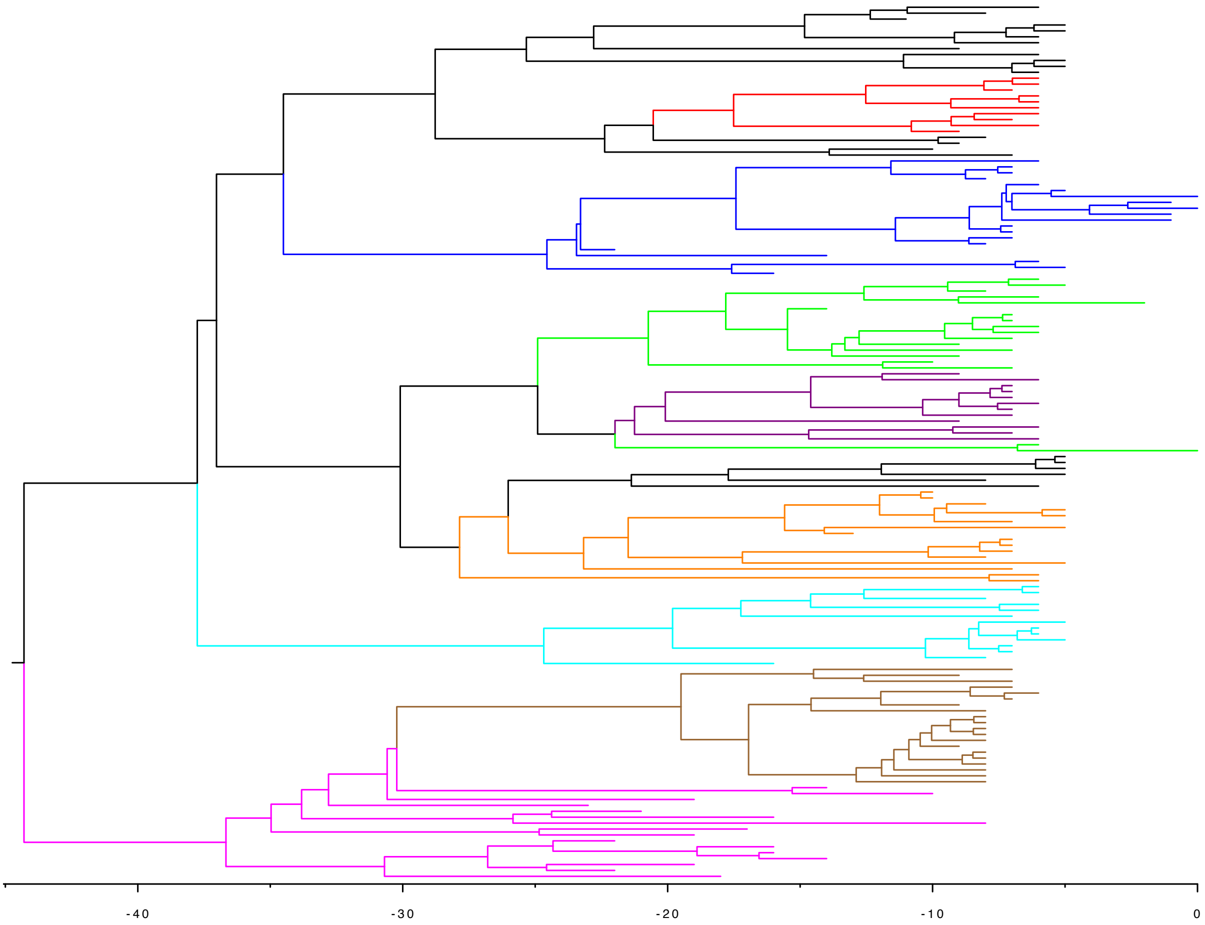

Supplementary Figure S4 – Skyline, strict clock, MCC tree. NI 1.140 lineage 15SNP clusters 2 (red), 4 (blue), 6 (green), 7 (orange), 8 (purple), 10 (turquoise), 11 (brown) and 12 (pink) - colour coded as per figure 3A.

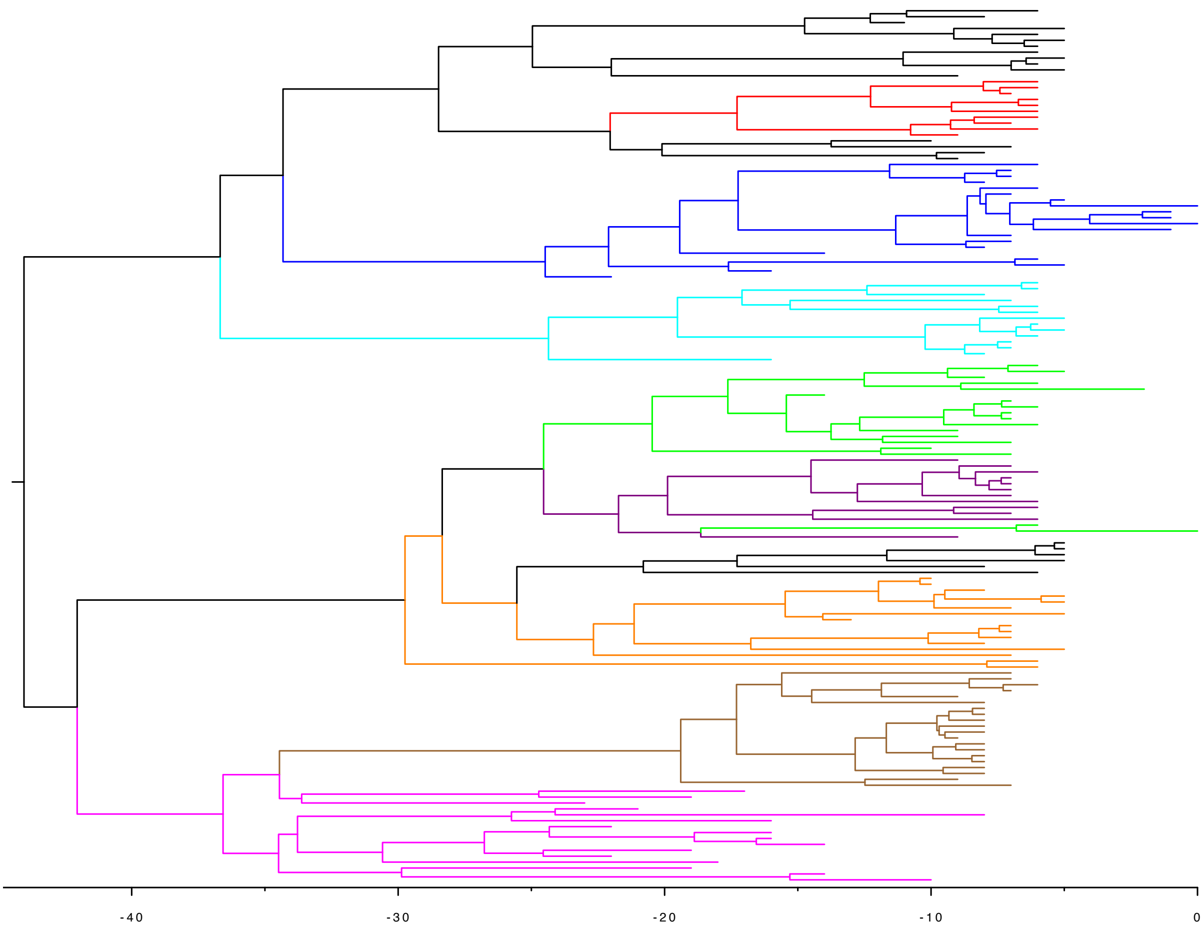

Supplementary Figure S5 – Skyline, relaxed clock, MCC tree. NI 1.140 lineage 15SNP clusters 2 (red), 4 (blue), 6 (green), 7 (orange), 8 (purple), 10 (turquoise), 11 (brown) and 12 (pink) - colour coded as per figure 3A.

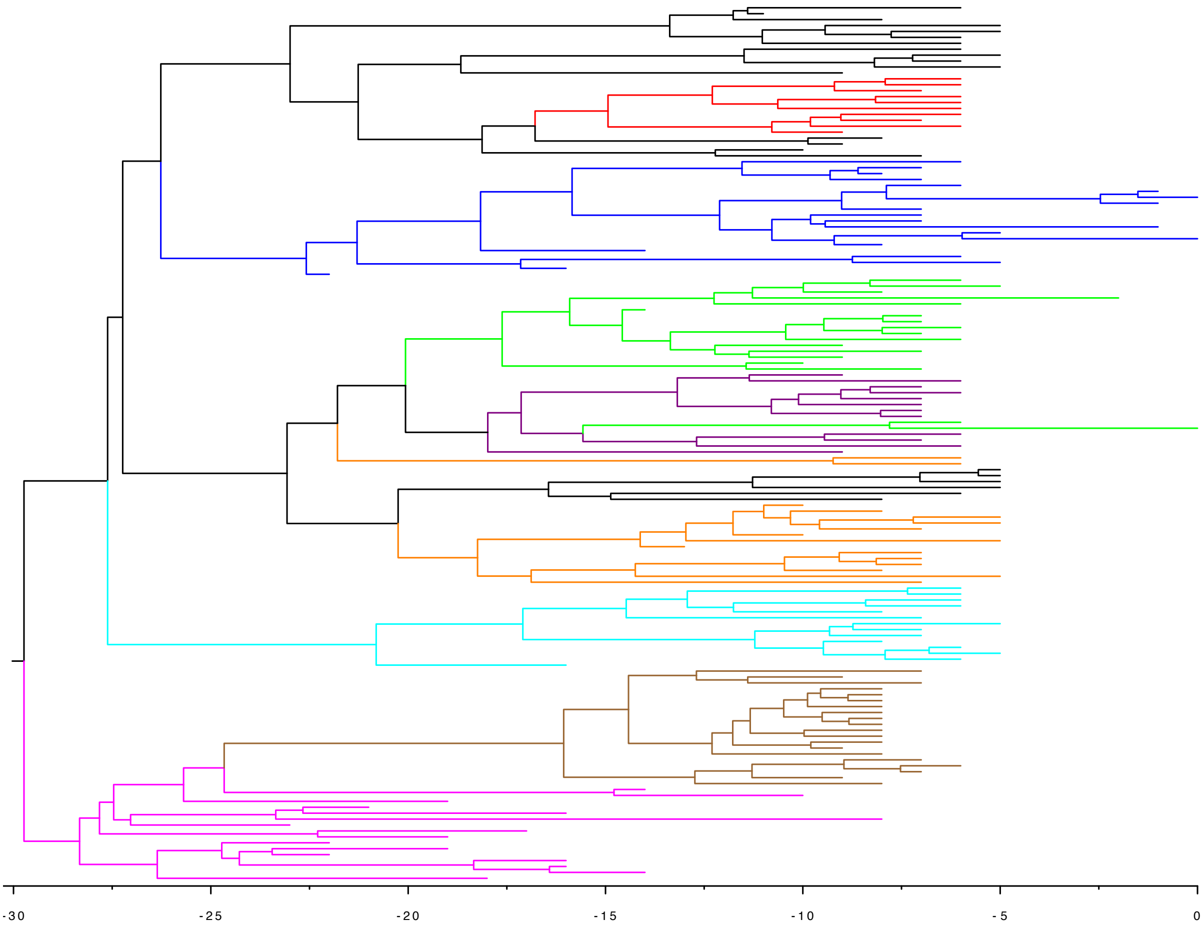

Supplementary Figure S6 – BDSky, strict clock, MCC tree. NI 1.140 lineage 15SNP clusters 2 (red), 4 (blue), 6 (green), 7 (orange), 8 (purple), 10 (turquoise), 11 (brown) and 12 (pink) - colour coded as per figure 3A.

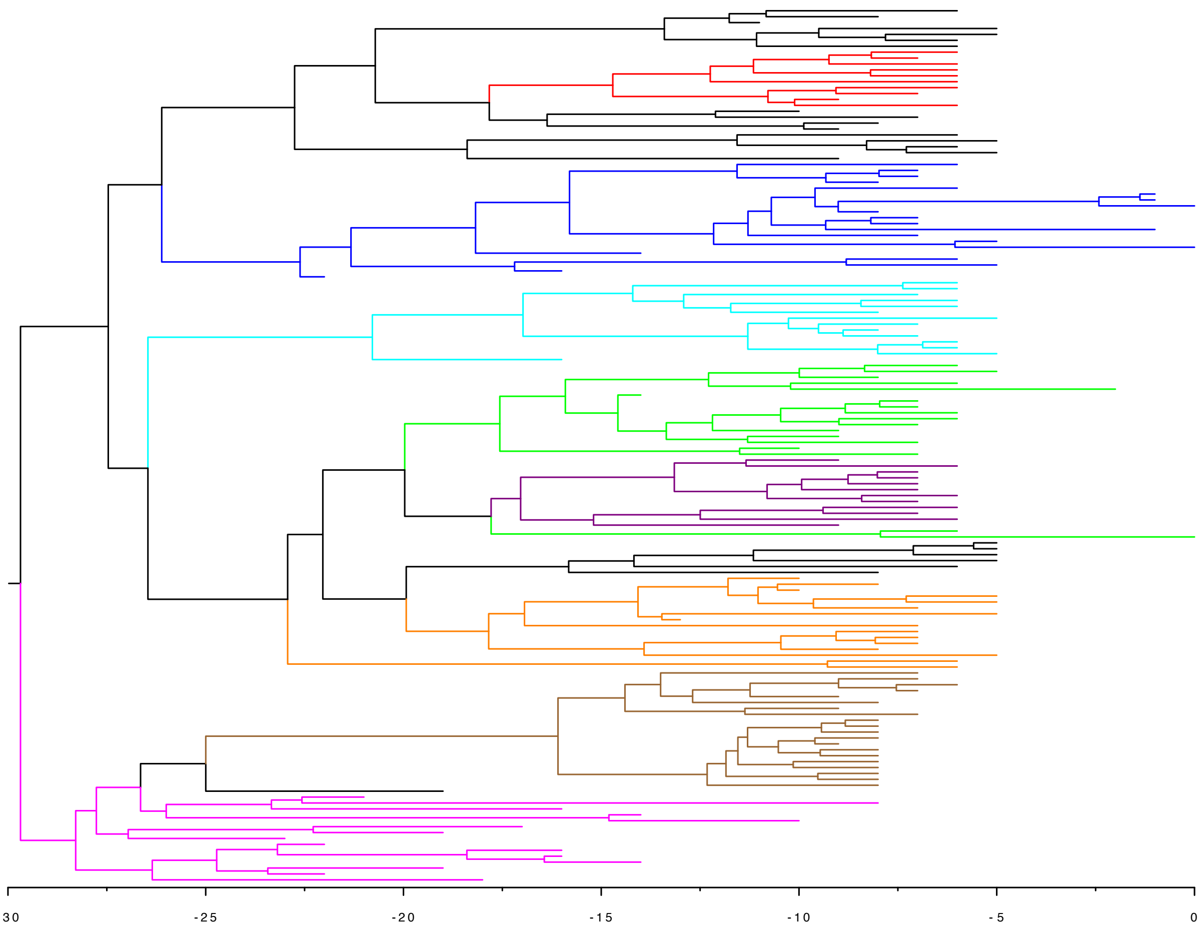

Supplementary Figure S7 – BDSky, relaxed clock, MCC tree. NI 1.140 lineage 15SNP clusters 2 (red), 4 (blue), 6 (green), 7 (orange), 8 (purple), 10 (turquoise), 11 (brown) and 12 (pink) - colour coded as per figure 3A.

Model	South NI clusters 11 and 12 tMRCA (yrs before present) plus 95% HPD	East NI clusters 2, 4, 7 and 10 tMRCA (yrs before present) plus 95% HPD	West NI clusters 6 and 8 tMRCA (years before present plus 95% HPD)
**Constant strict**	41.0 (32.4-50.6)	41.5 (31.0-53.6)	23.1 (17.7-29.0)
**Constant relaxed**	41.4 (32.4-51.8)	41.5 (30.5-53.7)	23.1 (17.7-29.4)
**Skyline strict**	37.3(30.1-46.2)	38.6 (29.5-49.4)	25.3(18.5-33.4)
**Skyline relaxed**	37.5 (29.7-45.5)	37.1 (28.9-47.4)	25.0 (18.2-33.0)
**BDSky strict**	28.4 (26.5-30.1)	27.6 (25.5-29.8)	20.2 (16.8-23.9)
**BDSky relaxed**	28.3 (26.3-30.0)	27.5 (25.2-29.8)	22.0 (16.5-23.8)

Supplementary Table 1 – Divergence times for southern eastern and western NI clusters from all six BEAST2 models.

	Constant strict	Constant relaxed	Skyline strict	Skyline relaxed	BDSky strict	BDSky relaxed
**Clade 2**	0.99	1.00	1.00	1.00	0.99	0.99
**Clade 4**	1.00	1.00	1.00	1.00	1.00	1.00
**Clade 6**	1.00	0.99	1.00	1.00	1.00	1.00
**Clade 7**	0.32	0.99	0.32	0.99	1.00	1.00
**Clade 8**	0.46	0.47	0.38	0.39	0.47	0.48
**Clade 10**	1.00	0.99	1.00	1.00	1.00	1.00
**Clade 11**	1.00	1.00	1.00	1.00	1.00	1.00
**Clade 12**	0.85	0.82	0.93	0.92	0.67	0.63

Supplementary Table 2 – Posterior support for the MRCA of the eight 15SNP defined WGS clades.
